# The Causes and Treatment of Ununited Fracture

**Published:** 1895-10-26

**Authors:** Wheelton Hind

**Affiliations:** Assistant Surgeon to the North Staffordshire Infirmary.


### Medical Progress and Hospital Clinics.

[*The Editor will be glad to receive offers of co-operation and contributions from members of the profession. All letters should be addressed to* The Editor, at the Office, 428, Strand, London, W.C.]

### THE CAUSES AND TREATMENT OF UNUNITED FRACTURE.

By Wheelton Hind, M.D., B.S.Lond., F.R.C.S., Assistant Surgeon to the North Staffordshire Infirmary.

During the last few years I have had to operate on three case of ununited fracture of long bones, once on the femur, twice on the tibia—but in these cases one was at the junction of the upper and middle thirds, the second at the junction of the lower and middle thirds. All cases were in men. After a brief summary of the causes and treatment of ununited fracture of bones, I shall proceed to give details as to the methods of procedure I adopted and the results.

Ununited fractures are due to two classes of causes. The one consists of general systemic causes; in the other class the causes are purely mechanical, and it is this class which furnishes as a rule the cases for operative treatment.

**Class I—Systemic Causes—Syphilis, Scurvy, Wasting Disease, Insufficient Food.**—Of these scurvy is the most potent. The others are almost hypothetical causes, which with others of the same class are generally known in our text-book accounts, in order to give examinees a chance of concocting an answer; but clinically these causes are seldom or never ob served.

**Class II—Mechanical Causes.**—1. Nonapposition of fragments, which may be subdivided into separation of ends, as in fractured patella, and oblique fracture and misplacement of fragments, so that the raw and bony surfaces are not in contact. 2. The interposition of muscle or fascia between the ends of the bone. 3. Necrosis of the ends of the bone in compound fractures. 4. Rupture of the nutrient artery, the fracture being near the point of entrance. The latter is a doubtful cause, as the callus-forming material is thrown out by periosteum and other tissues in the neighbourhood of the bone to a very large extent. 5. Imperfect blood supply to the limb, owing to concomitant damage to the main vessels. 6. Mobility of the fragments.

**Treatment.**—The treatment of ununited fractures obviously falls into two categories. First, general, which may be dismissed in a few words. The general health and nutrition of the patient should be looked after, and the specific treatment for scurvy or syphilis be perseveringly followed.

The local treatment has for its object first, rest and the proper apposition of the ends of the fragments; second, that the ends should be rendered immobile; third, that endeavours should be made to excite callus formation. To this end tenotomy, careful splinting, rubbing the ends of the bones together, counter-irritation by blistering, friction, or seton. Failing these, more active measures may be adopted, such as subcutaneous incision or the driving of ivory pegs into the ends of the bones to cause a certain amount of irritation, simple resection, resection with pegging ligature or screwing, and finally bone grafting after resection. These, then, are the various means at our disposal, one or other of which may be selected according to the suitability of the case. In the case of necrosis of the ends of the fragments, nothing remains but to remove the dead tissue either by resection, which I have seen successfully carried out in the tibia, or by waiting for pathological processes to separate the necrosed portions.

The first case on which I had to operate was one of a bad oblique fracture of the lower end of tibia and fibula. The accident happened on January 1st, 1892. It had been treated for several months by rest and splints, but although the leg was fairly firm, there was no bony union, and the leg would bear no weight. The patient came from a neighbouring workhouse union, and was fifty years of age. On November 7th, 1892, with every aseptic precaution an incision was made for about 4 inches in length over the seat of the fracture, and the bone was cut down upon, and the tissues on all sides were freed by a blunt dissector and raspatory, all extraneous callus, of which there was a good deal, being removed by bone forceps. It was then seen that the fracture had been very oblique, and that the fragments had become displaced upwards and downwards. The fibrous union was then broken down and the ends of the bones freed, so that they could be drawn down—the most difficult part of the procedure, for it necessitates detaching all the tissues round each fragment. Then I carefully resected the ends of each bone, using first a saw, and afterwards bevelling each bone with chisel and mallet, so that the foot came into its proper position, and removed the sharp projecting angle of each bone. Then, with a drill, holes were made obliquely in each fragment, and a thick soft silver wire was passed and firmly twisted up, the ends being hammered into the bone. All fragments and bone chips were then carefully removed, and the toilet of the wound completed and the skin united by incisions without drainage. The subsequent history of the case was simply that the wound healed by first intention, and the patient left the hospital on December 15th, 1891, and when I saw the man last, some few months after operation, there was complete bony union, but he had not then recovered good use, owing to long interference with the muscle nutrition. The fibula caused no trouble in bringing the ends of the tibia into complete apposition.

Case 2 was much more formidable. It occurred in a canal boatman, aged forty, who had lost one leg just above the knee, and who came in with a fractured femur about the middle of the thigh, on June 21st, 1894. The leg was a very thick and muscular one, and there was great displacement and bruising. After four months' treatment it was decided to operate. An incision six inches long was made on the outer side of the left thigh, and continued through the muscle. The leg was enormously swollen and full of callus, and it was a matter of the utmost difficulty to chisel this away and bring the fragments together. The lower fragment had passed behind and outside the upper one for some inches, and each end was buried in a large mass of callus, but there was only fibrous union between the periosteal surfaces where they were in contact.

After one and a half hour's hard work the bones were resected and dragged into apposition, and it was only after removing at last one and a half inches from each fragment that they could be got into line. All extraneous callus was removed, and the ends were brought as well as possible into contact, but, owing to the expanded and transversely enlarged ends, they could not be very accurately adapted. The ends were secured in this position by thick silver wire and the wound washed out and sewn up, dressed, and the leg splinted. All went well at first and the wound united, to break down at the end of a fortnight with free suppuration. This condition of things went on for some time and gradually subsided, leaving a small sinus, the leg gradually becoming firmer, till at the end of eight months there was firm bony union. A small sinus still remained, probably leading down to the wire suture, which was explored on October 17th last, and the wire easily removed, when the sinus immediately began to close. It was a very important thing in this case to save the limb, owing to the loss of the other leg. The man can now get about a bit with the aid of crutches, which are necessary on account of his wooden leg.

Case No. 3 was a fracture of the left tibia, and was the easiest operation of the three. The patient was a man of thirty-five, who met with his accident on October 11th, 1894, and though the leg was in fair position, there was not a great deal of callus thrown out, and very little deformity. On March 5th, 1895, I operated by a U-shaped flap and exposed the tibia well, finding a very oblique fracture, the fragments separated by fibrous tissue. Freeing the end of the fragments and dividing the fibrous connections, I took obliquely from above downwards and without inwards, sections from each fragment with a saw, and carefully bevelling the faces with a chisel and removing surrounding masses of callus which interfered with perfect apposition I got the bones well in contact by the whole length of my section. I then drilled a hole and inserted one of Lane's screws, two inches long, which I drove home. The toilet of the wound was made and the wound closed. The case did well, nearly the whole wound healing by first intention; a smallsinus, however, was left leading down to the screw, the man leaving the hospital in a plaster splint, but the bony union seemed quite firm.

This class of operations is by no means easy. There is great difficulty in clearing the ends of the bones, especially when working by a single long incision, it being very difficult to free the bone from its callus and attachments behind and at the opposite side from the incision. A flap incision where possible gives much greater scope for operative procedure.

The points I regard as important are to remove as much surrounding callus as possible. It has been thrown out in wrong directions and is useless, and impedes muscular action during convalesence; and, indeed, is so often in the way of getting the fragments into line that the removal is a necessity, and further the complete removal of the fibrous union is essential. Complete adaptation of the ends of the fragments is not essential as long as they do come into contact at some points; but, other things being equal, the more completely the refreshed surfaces of the fragments are in apposition he more rapidly union takes place.

Oblique fractures are suitable for pegging or for adapting with ligatures or screws, or they will do well if only the surfaces can be kept in contact by accurate splinting and pressure. In uniting the patella I have on three occasions used silk or catgut ligatures with perfect results. The precise method of securing union must depend on the nature of each case. In only one of my three cases has the material used caused any irritation; and in the thigh case until the wire was removed.

The after-treatment has for its object the restoration of muscular power, so diminished by injury and disuse. To this end systematic massage is a great help, and daily movements of the joints which have become stiffened during treatment.

